# The CARE (CAse REport) guidelines and the standardization of case reports

**DOI:** 10.1186/1752-1947-7-261

**Published:** 2013-11-27

**Authors:** Richard A Rison, Michael R Kidd, Christian A Koch

**Affiliations:** 1 Presbyterian Intercommunity Hospital Health Stroke Program, University of Southern California Keck School of Medicine, Los Angeles County Medical Center, 12401 Washington Boulevard, Whittier, CA 90602, USA; 2 Faculty of Health Sciences, Flinders University, GPO Box 2100, Adelaide SA 5001, Australia; 3 Department of Medicine, G. V (Sonny) Montgomery VA Medical Center, Jackson, MS 39216, USA; 4 Division of Endocrinology, Department of Medicine, University of Mississippi Medical Center, Jackson, MS 39216, USA

## Abstract

Case reports comprise the core of *Journal of Medical Case Reports*, are a time-honored tradition firmly established within the medical literature, and represent a growing importance of valuable clinical medical information in our modern information-flowing times. While there is already a body of published literature on how and when to write a case report and both *Journal of Medical Case Reports* and BioMed Central make known their own criteria, case report quality across all of the medical literature is still variable. Additionally, although health reporting agencies do have standardization guidelines for other aspects of health-care reporting, there has never been an organizational body responsible for international standardization of how to write a case report. With the newly-published CARE (CAse REport) guidelines, Gagnier and colleagues hope to change this. This editorial serves as a brief introduction to the CARE guidelines and briefly examines the proposed standardization of case reports. We invite feedback on the CARE guidelines from all of our readers and encourage their trial run implementation by our own case report authors.

## 

In the immortal words of the famous Canadian physician, first Professor of Medicine and founder of the Medical Service at Johns Hopkins Hospital, and of course our ‘Father of Modern Medicine’, William Osler (July 12, 1849 to December 29, 1919):

‘Always note and record the unusual…Publish it. Place it on permanent record as a short, concise note. Such communications are always of value.’ [1].

But how exactly do we do this? What makes a good case report? While most of us may follow the maxim ‘I know it when I see it’, few if any have had formal training on what constitutes a good case report and how to write one. We are initially exposed to case reports in our readings during medical training, and many of us write our first ones in residency under the auspices of one of our attending professors. Unlike many other aspects of our medical training, that of writing case reports is not standardized. There is a wide body of published literature on how to construct a case report, including the different sections of a case report (abstract, introduction, case presentation, and so on) and when to write one [2,3], but different journals have different criteria and case report quality can be variable [4,5].

The consensus-based clinical case report guideline development paper by Gagnier and colleagues now addresses the important issue of standardizing case report formats [6]. The CARE (CAse REport) guidelines consist of a three-phase consensus-based process that involved 27 participants, all of whom have involvement in clinical case report writing and editing (including the authors of this editorial). The process included telephone interviews, a face-to-face meeting and, finally, a refined checklist of 13 items that should comprise a case report. Throughout the entire process, we were all asked what information was required in case-reporting guidelines, the rationale for our suggestions, and references that supported our reasoning. The primary items of the CARE checklist, which should be familiar to our readers, are the following: title, key words, abstract, introduction, patient information, clinical findings, timeline, diagnostic assessment, therapeutic interventions, follow-up and outcomes, discussion, patient perspective, and informed consent. The manuscript provides a useful table displaying the CARE guidelines checklist that authors can easily refer to.

The CARE guidelines have been presented at international conferences and workshops where they have been met with enthusiasm. These included the Peer Review and Biomedical Publication Congress in Chicago sponsored by the *British Medical Journal* and the *Journal of the American Medical Association* on September 10, 2013, which coincided with simultaneous publication within the following medical journals: *BMJ Case Reports*, *Deutsches Ärzteblatt, Headache, Global Advances in Health and Medicine, Journal of Clinical Epidemiology, Journal of Dietary Supplements* and the *Journal of Medical Case Reports*. Additionally, by involving *Journal of Medical Case Reports* and BioMed Central (BMC), there will be open-access outreach to the 250 journals depositing case reports into the BMC *Cases Database*.

As Gagnier and colleagues aptly point out, there are already multiple organizations for health-care reporting agencies including for the following publication types: randomized controlled trials (Consolidated Standards of Reporting Trials, or CONSORT) [7], observational studies (Strengthening the Reporting of Observational studies in Epidemiology, or STROBE) [8], and systematic reviews and meta-analyses (Preferred Reporting Items for Systematic Reviews and Meta-Analyses, or PRISMA) [9]. Guidelines have also been developed for adverse-event case reports [10], and all authors should be familiar with the Committee on Publication Ethics (COPE) [11] and the **E**nhancing the **Qua**lity and **T**ransparency **O**f Health **R**esearch (EQUATOR) Network [12]. Up until the CARE guidelines publication, however, general international reporting guidelines for case reports have not existed.

Readers of *Journal of Medical Case Reports* (launched by BMC in 2007) recognize the time-honored importance of case reports as a valuable source of new ideas and information in clinical medicine, and there are published editorials available on the continued importance of open-access case reports in our modern information-flowing world [13-15]. When BMC’s open-access *Cases Database* was created in 2012 by author MRK and colleagues, it brought the total number of published case reports to 11,000, from 50 included medical journals. This grew to more than 26,000 case reports from 212 included medical journals within the span of just six months. The data continues to grow: 28,298 peer-reviewed medical case reports from 250 journals upon last check (!) [16]. BMC has also now published personalized advice on how to write a case report for *Journal of Medical Case Reports* and *BMC Research Notes* along with an inside look into the editorial process [17].

We hope that the CARE guidelines will provide an international, general, non-journal-specific framework for completeness and transparency for published case reports, striking a balance between adequate detail and concise writing that can be used by all authors for all journals that publish case reports. The CARE guidelines are available on a dedicated website [18] along with the EQUATOR Network website [12], and will be translated into multiple languages. All authors, journal editors and peer reviewers are encouraged to pilot the CARE checklist and provide feedback that can be incorporated into regular updates. Research into the impact of the CARE guidelines on case reporting will also be conducted, and we look forward to a follow-up article showing the impact on clinical case reports. We also invite comments from all BMC case report readers, authors, editors and peer reviewers on what you think and how you feel that the CARE guidelines can be incorporated into not just *Journal of Medical Case Reports* but all BMC journals that publish case reports, including *BMC Research Notes* and the rest of the BMC series.
